# Quality of life after melphalan percutaneous hepatic perfusion for patients with metastatic uveal melanoma

**DOI:** 10.1097/CMR.0000000000000947

**Published:** 2023-12-01

**Authors:** Ganesh Vigneswaran, Weeratunge Malalasekera, Victoria Smith, Tom Gibson, Shian Patel, Matthew Wheater, Ioannis Karydis, Sanjay Gupta, Brian Stedman, Sachin Modi

**Affiliations:** a Department of Interventional Radiology, University Hospital Southampton NHS Foundation Trust; b Department of Cancer Sciences, University of Southampton; c Department of Oncology; d Department of Anaesthesia, University Hospital Southampton NHS Foundation Trust, UK

**Keywords:** melphalan percutaneous hepatic perfusion, quality of life, uveal melanoma

## Abstract

**Background:**

Recent studies indicate that melphalan percutaneous hepatic perfusion (M-PHP) for liver metastases from ocular melanoma (mUM) improves survival. Importantly, this benefit must be carefully balanced with changes in a patient‘s quality of life (QoL). This study examines the QoL changes post-M-PHP.

**Methods:**

Retrospective analysis of the change in QoL using the Functional Assessment of Cancer Therapy-General (FACT-G) with mUM patients receiving M-PHP (*n* = 20). The FACT-G scores, which comprise physical (PWB), social (SWB), emotional (EWB) and functional (FWB) wellbeing were measured pre-procedure and at day 1, day of discharge (mean = 2.4 days), 7, 14 and 28 days after M-PHP therapy. Wilcoxon signed-rank test gauged QoL domain changes.

**Results:**

Baseline FACT-G median (IQR) scores were 101.8 (21.8). QoL scoring significantly decreased immediately after the procedure [day 1; 85 (27.5); *P* = 0.002] and gradually improved over time. By day 28, QoL almost returned to pre-procedure levels [100.3 (13.8); *P* = 0.31]. Subscore analysis revealed that the initial drop in QoL at day 1 post-procedure was attributable to the PWB (28 vs. 24; *P* = 0.001) and FWB domains (26 vs. 18.5; *P* < 0.001). By day 28 there was a statistically significant improvement in EWB (*P* = 0.01).

**Conclusion:**

QoL following M-PHP decreases immediately after therapy and is not significantly different from baseline by the day of discharge. By day 28 there is improved emotional well-being. This study could help to optimize the time between treatment cycles when combined with toxicity data and blood count recovery.

## Introduction

Uveal melanoma (UM), while a relatively uncommon form of melanoma, is the most common intraocular malignancy of adulthood [1]. Metastatic spread is seen in 25–34% of patients following successful initial local treatment [2,3]. Median survival in patients with metastatic disease is extremely poor at 1 year [3]. However, this is worse in cases of hepatic metastases (the most common site of metastasis [4]) where median survival can be <6 months [5,6]. This is despite the fact that many patients only have a single-site disease at diagnosis [4].

Liver-targeted therapies are a promising treatment for those with liver-only mUM, whether as monotherapy [7] or in conjunction with immunotherapy [8]. Melphalan percutaneous hepatic perfusion (M-PHP) provides the opportunity to expose the liver to high-dose chemotherapy without exposing peripheral non-target tissues to dangerous doses, while also exploiting the relative hyperperfusion of tumours compared with normal liver parenchyma [7]. This is achieved by introducing melphalan through an arterial catheter in the hepatic artery and then simultaneously filtering the hepatic venous blood via a double-balloon catheter positioned in the hepatic inferior vena cava [9]. Multiple studies have shown both a favourable safety profile for the procedure—in terms of peripheral non-target melphalan dose and clinical complications—and promising results in terms of radiological response and survival parameters [7,10–13].

An attempt to measure the impact of the procedure on patient quality of life (QoL) has only been undertaken in a few cases [10,14], creating a knowledge gap limiting our ability to weigh up the survival benefits of M-PHP against any QoL effects. In this study, we aim to address this by showing the results of a single-center study evaluating the temporal change in the QoL after M-PHP treatment.

## Materials and methods

To measure QoL, the Functional Assessment of Cancer Therapy-General (FACT-G) questionnaire was used. This is a 27-item questionnaire that measures four separate subdomains of QoL; physical (PWB), social (SWB), emotional (EWB) and functional (FWB) wellbeing. It has been specifically developed for and validated in oncology patients [19].

Data were collected from a subset of patients who underwent M-PHP therapy at our institution between August 2020 and January 2023 (*n* = 20). This patient cohort was comprised of a subset of a larger group, the survival data for whom has been analysed in a previous study [7] and seven further patients treated thereafter with QoL assessments. M-PHP therapy is administered as a general anaesthetic procedure with 3 mg/kg melphalan delivered via a Hepatic CHEMOSAT Delivery System (Delcath Systems, Inc., New York, USA). A full description of the procedure and the selection criteria for the overall group can be found in the *Materials and methods* section of this study [7]. Patients were asked to complete FACT-G questionnaires pre-procedure and at day 1, day of discharge [mean = 2.4 days (2–3)], day 7, 14 and 28 post-procedure. Questionnaires were added to the standard of care from August 2020, and all consecutive patients who consented to the questionnaire were used for this analysis. Any patient for whom full questionnaire responses were not available were excluded from the analysis (*n* = 0).

All statistical analysis was performed using MATLAB 2021 (Mathworks Inc, Natick, Massachusetts, USA). Analysis was performed on the change in FACT-G score from pre-procedure to other time points, with further subgroup analysis performed on the four QoL subdomains. Statistical testing was performed between pre-procedure FACT-G scores and scores at subsequent time points using a Wilcoxon signed-rank test (*P* < 0.05).

## Results

A total of 20 patients underwent M-PHP therapy with an assessment of QoL metrics described in the methods. The baseline characteristics of this patient cohort are displayed in Table 1.

**Table 1:** Baseline characteristics and outcomes

Patient characteristics		Mean (std)	N and (%)
Age		56.8 (16)	
Gender	Female		16 (80)
	Male		4 (20)
Performance status	0		10 (50)
	1		8 (40)
	2		2 (10)
Disease extent at treatment	Intrahepatic		19 (95)
	Intrahepatic + extrahepatic		1 (5)
Disease burden in the liver	High (>10 lesions or >50% liver involvement)		10 (50)
	Low		10 (50)
Complication	Hepatic artery dissection		1 (5)
	Mild toxicity		1 (5)
	None		18 (90)
Best liver response by RECIST1.1 criteria	CR		5 (25)
	PR		11 (55)
	SD		3 (15)
	PD		1 (5)

CR, complete response; PR, partial response; PR, progressive disease; SD, stable disease.

**Fig. 1:**
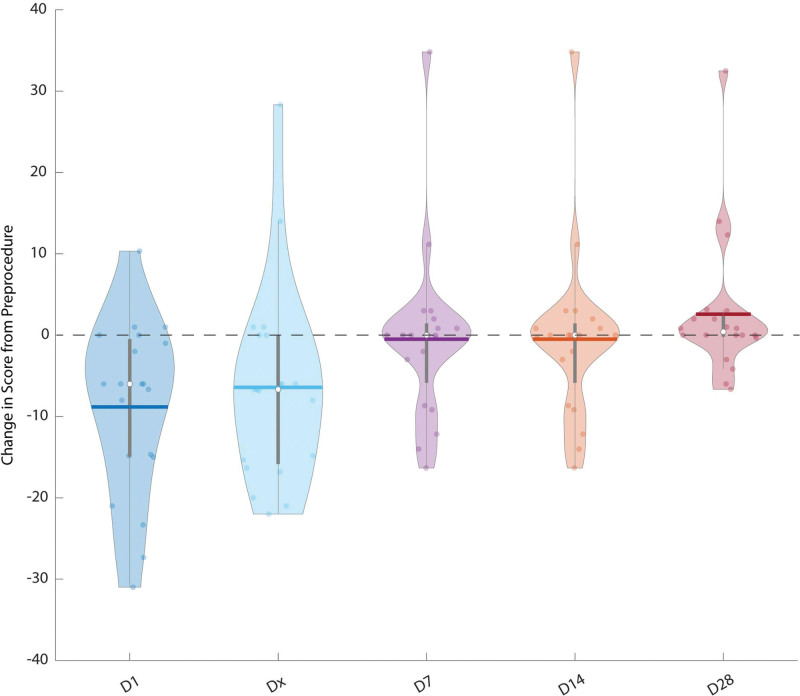
Violin plots showing the difference in the overall FACT-G QoL score compared with baseline at day 1 (D1), day of discharge (Dx), day 7 (D7), day 14 (D14) and day 28 (D28) compared to pre-procedure. Each plot shows the mean (thick horizontal lines), median (white dots) and standard deviations (thick grey vertical lines) across the patient group (*n* = 20). FACT-G, Functional Assessment of Cancer Therapy-General; QoL, quality of life.

The baseline FACT-G score median (IQR) was 101.8/108 (21.8). Compared to this baseline, QoL scoring significantly decreased immediately after the procedure [day 1; 85 (27.5); *P* = 0.002]. This gradually improved over time (Fig. 1), although at the point of discharge, there remained a statistically significant difference between pre- and post-treatment scores 87.8 (20.9), c.f. baseline *P* = 0.02. By day 14, there was no residual significant difference compared to the pre-procedure score [95 (18); *P* = 0.57] and by day 28, overall scores were almost at baseline [100 (13.8); *P* = 0.31].

**Fig. 2:**
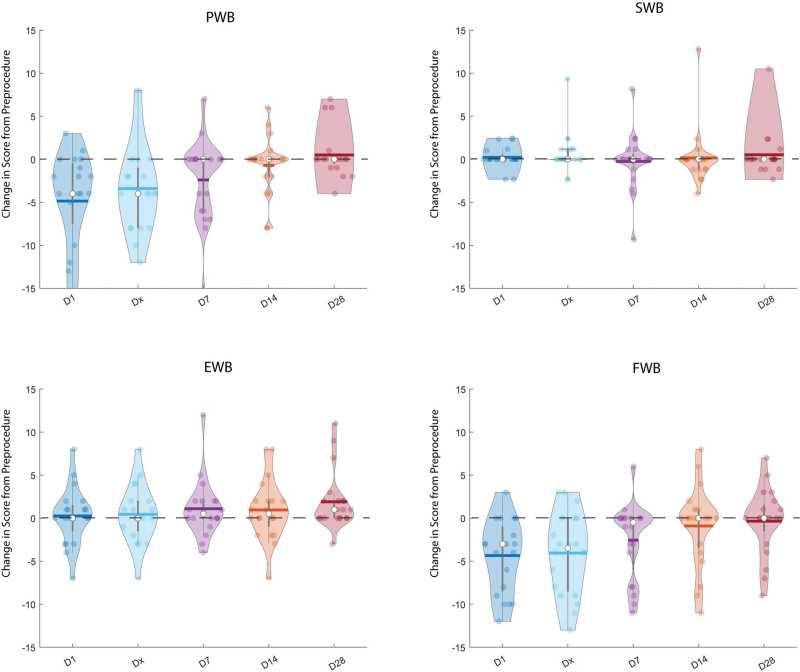
Violin plots showing the difference in the physical (a), social (b), emotional (c) and functional (d) subscore domains at day 1 post-procedure, day of discharge, day 7, day 14 and day 28 (left to right on x-axis). Each plot shows the mean (thick horizontal lines), median (white dots) and standard deviations (thick grey vertical lines) across the patient group (*n* = 20).

To better understand any underlying causes, subscore analysis was performed (Fig. 2). This revealed that most of the initial drop in QoL at day 1 post-procedure was attributable to the PWB [pre-procedure: 28 (1) vs. post-procedure: 24 (9); *P* = 0.001] and the FWB domains [pre-procedure: 26 (9.5) vs. post-procedure: 18.5 (10); *P* < 0.001]. Again, these scores returned to baseline over the subsequent time points. Interestingly, by day 28 there was a statistically significant improvement in EWB domain compared to baseline (pre-procedure:20.5 vs. post-procedure: 22; *P* = 0.01). The other subgroups showed no statistically significant differences at day 28.

## Discussion

UM with hepatic metastases represents a disease entity associated with significant mortality and morbidity, with few established systemic or local therapies that significantly improve survival. This has led to an array of potential treatments being developed in recent years. While these may provide some survival benefits, it is important to weigh these against any negative impact on QoL for the patient, which may negate the perceived value of any improved life expectancy. In this analysis, we sought to assess this by specifically looking at the impact of M-PHP therapy on a patient’s QoL.

We found that although there is a small reduction in QoL scores immediately post-procedure, these normalise with time and are not statistically different from baseline by day 7 post-procedure. Subscore analysis showed that this initial drop in QoL was driven by physical and functional well-being factors. Although, unsurprisingly, this objectively indicates the procedure has an initial physical and functional toll on the patient. Another interesting result from our study is that patients report an increased emotional well-being at day 28 compared to baseline. This may reflect increased optimism regarding the course of their disease or may suggest improvement in other physical symptoms that are not captured in the physical well-being sub-score.

To our knowledge, no previous study has assessed QoL in the same peri-procedural time frame as has been conducted in this study. In their study, Vogl *et al*. collected QoL data for six patients pre-procedure and at 6 weeks post-procedure using the EORTC QLQ-C30 tool [14]. They reported a qualitative improvement in QoL at 6 weeks compared to pre-procedure. In comparison, our data is collected from a greater number of patients and at multiple post-procedure time points allowing us to better understand the temporal evolution of QoL. Although we do not demonstrate a significantly increased overall QoL following the procedure, this may be because our follow-up interval was insufficient to see this and would be unexpected for a disease process where no cure is anticipated.

Impact on QoL has also been assessed with other novel systemic therapies. For example, Atkinson *et al*. [15] have looked at the correlation between objective drug toxicity and subjective patient QoL in those given selumetinib (a MEK inhibitor) for mUM compared to those randomised to standard chemotherapy. Interestingly, they found no statistical difference between baseline and post-treatment QoL for selumetinib or standard therapy. A not-dissimilar study by Mouriaux *et al*. [16] also shows that at later time points (24 weeks), QoL in these patients appears decreased.

As more evidence is unveiled from large multicentre clinical trials, we are optimistic that additional evidence will support our findings that M-PHP therapy has minimal effects on QoL and procedure-related adverse events. The SCANDIUM trial serves as a case in point. Early findings released in 2022 [17,18] revealed serious complications in 19.5% of patients undergoing the treatment for the first time vs. 6.5% in controls. Additionally, we eagerly anticipate the forthcoming publication of results from the international FOCUS study.

### Conclusion

Novel systemic and targeted therapies hold much hope for the improved survival and QoL of patients with mUM. In our analysis of patients undergoing M-PHP therapy, we have added to the evidence that suggests that it is well tolerated by patients making any associated improvement in mortality all the more valuable.

## Acknowledgements

We would like to thank Pippa Woodcock for her contribution to this project.

Conceptualisation: S.M., B.S. Methodology: G.V., W.M., M.W., I.K., S.G., B.S., S.M. Data collection: V.S., T.G., S.P., M.W., I.K., S.G., B.S., S.M. Data analysis. G.V., W.M. Writing–review and editing: G.V., W.M., M.W., I.K., S.G., B.S., S.M.

### Conflicts of interest

S.M., B.S., and S.G.: consultancy contract and Honoraria with Delcath. I.K. and M.W.: Honoraria with Delcath. For the remaining authors, there are no conflicts of interest.
